# Supplementary material for: Microbial Consortia and Mixed Plastic Waste: Pangenomic Analysis Reveals Potential for Degradation of Multiple Plastic Types via Previously Identified PET Degrading Bacteria
Source: Int J Mol Sci. 2022 May 17;23(10):5612. doi: 10.3390/ijms23105612 (PMC9146961; doi:10.3390/ijms23105612)
Supplement: Supplementary file 1 [file ijms-23-05612-s001.zip › Figure S3.pdf]

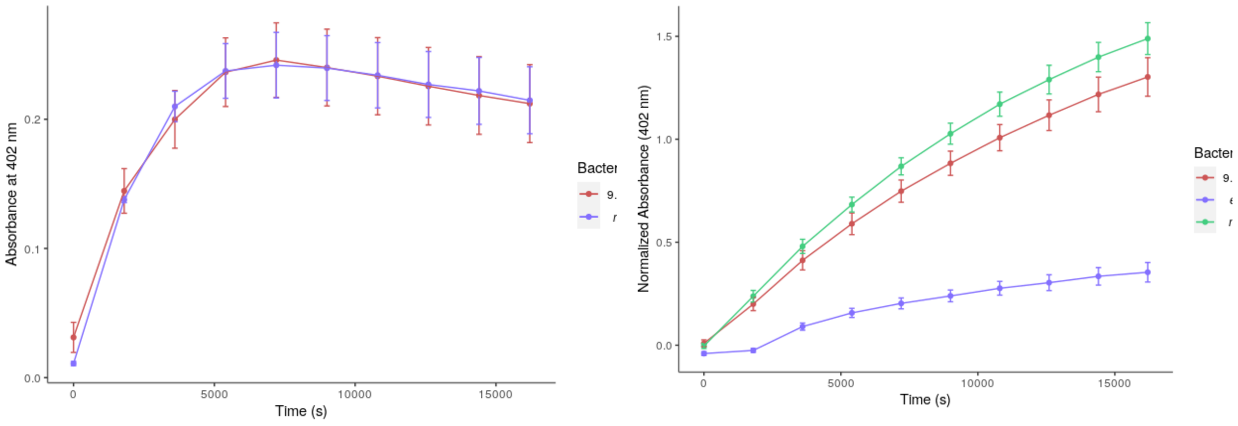

(**a**) (**b**)

Figure S3. Deletion of NlhH resulted in no significant loss in esterase activity against p-np-butyrate unlike EstB; (**a**)*ΔnlhH* on np-butyrate compared to wildtype control. (**b**) *ΔnlhH* and *ΔestB* esterase activity on p-np-butyrate compared to wildtype control.
