# Supplementary material for: Microbial Consortia and Mixed Plastic Waste: Pangenomic Analysis Reveals Potential for Degradation of Multiple Plastic Types via Previously Identified PET Degrading Bacteria
Source: Int J Mol Sci. 2022 May 17;23(10):5612. doi: 10.3390/ijms23105612 (PMC9146961; doi:10.3390/ijms23105612)
Supplement: Supplementary file 1 [file ijms-23-05612-s001.zip › Table S1.pdf]

Table S1: Optical Density of each sample type for Biolog Ecoplates after 24 hours at room temperature

Carbon Source	9.1	9.2	10	13.1	13.2	FC
L-Asparagine	0.015	1.570	1.222	0.670	1.429	1.586
D Galacturonic Acid	0.024	0.027	0.485	0.029	1.102	1.167
Putrescine	0.000	0.851	0.836	0.458	1.199	1.079
Tween40	0.018	0.865	0.888	0.325	0.838	1.010
L-Serine	0.031	1.003	0.886	0.421	0.412	0.966
γ -Aminobutyric acid	0.023	0.719	0.706	0.487	0.855	0.873
L-Arginine	0.000	0.548	0.420	0.296	0.708	0.866
D-Mannitol	0.017	0.845	0.563	0.320	0.101	0.843
4-Hydroxy Benzoic Acid	0.000	0.461	0.668	0.237	0.669	0.679
D-Galactonic Acid γ -Lactone	0.000	0.630	0.414	0.269	0.145	0.651
D Glucosaminic Acid	0.015	0.670	0.606	0.193	0.067	0.641
Pyruvic Acid Methyl Ester	0.022	0.536	0.508	0.295	0.571	0.618
Tween80	0.047	0.365	0.417	0.034	0.764	0.577
N-Acetyl Glucosamine	0.054	0.320	0.339	0.250	0.069	0.433
1-Glycerol phosphate	0.004	0.272	0.313	0.252	0.008	0.304
Phenylethylamine	0.010	0.000	0.051	0.013	0.463	0.242
D-Xylose	0.067	0.202	0.192	0.081	0.079	0.151
D-Cellobiose	0.034	0.171	0.046	0.056	0.031	0.079
Glycyl-L Glutamic Acid	0.020	0.229	0.061	0.045	0.039	0.078
Glycogen	0.043	0.065	0.068	0.066	0.079	0.058
L-Threonine	0.020	0.095	0.196	0.026	0.053	0.054
α-Keto Butyric Acid	0.026	0.059	0.036	0.036	0.000	0.038
L Phenylalanine	0.014	0.087	0.055	0.021	0.108	0.033
D-Malic Acid	0.000	0.046	0.096	0.021	0.026	0.020
β-Methyl-D Glucoside	0.021	0.050	0.013	0.000	0.000	0.017
Itaconic Acid	0.020	0.027	0.001	0.020	0.004	0.005
Glucose-1- Phosphate	0.028	0.023	0.010	0.021	0.026	0.000
α-D-Lactose	0.000	0.036	0.035	0.000	0.000	0.000
Erythritol	0.016	0.005	0.003	0.015	0.038	0.000
α- Cyclodextrin	0.009	0.319	0.007	0.000	0.027	0.000
2-Hydroxy Benzoic Acid	0.000	0.000	0.000	0.000	0.000	0.000
