# Supplementary figures and images for: Microbial Consortia and Mixed Plastic Waste: Pangenomic Analysis Reveals Potential for Degradation of Multiple Plastic Types via Previously Identified PET Degrading Bacteria
Source: Int J Mol Sci. 2022 May 17;23(10):5612. doi: 10.3390/ijms23105612 (PMC9146961; doi:10.3390/ijms23105612)

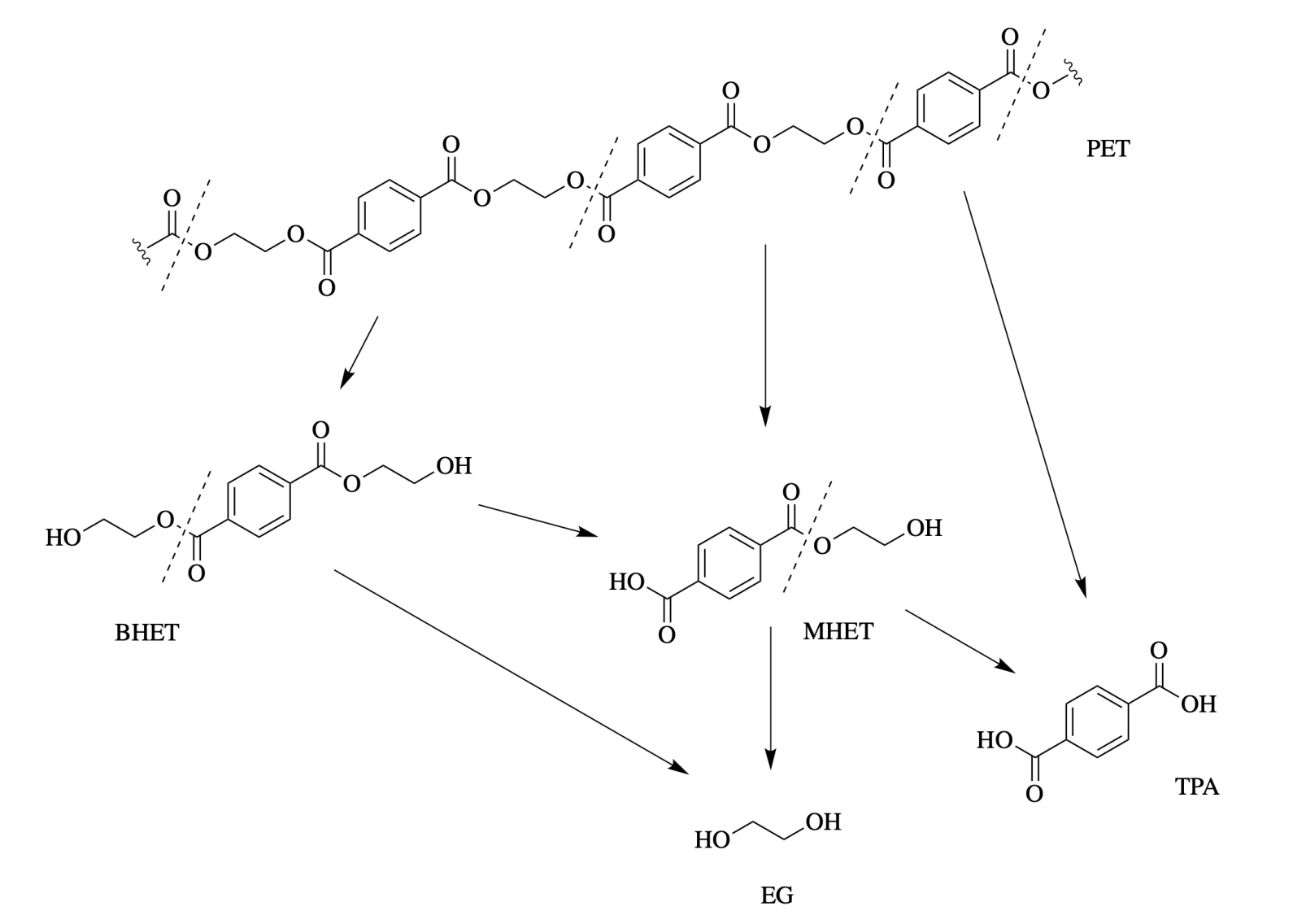

Supplement: Supplementary file 1 [file ijms-23-05612-s001.zip › Figure S2.png]
